# Factors influencing early recurrence of atrial fibrillation among elderly patients following radiofrequency catheter ablation and the impact of different antiarrhythmic regimens

**DOI:** 10.3389/fmed.2024.1393208

**Published:** 2024-06-27

**Authors:** Changdong Fei, Caitong Zhao, Yan Ma, Yupeng Liu, Renzheng Chen, Hualin Zhang

**Affiliations:** ^1^Department of Health Management Center, The 967th Hospital of Joint Logistics Support Force of Chinese PLA, Dalian, China; ^2^Department of Quality Control, General Hospital of Northern Theater Command, Shenyang, China; ^3^National Clinical Research Center of Geriatric Diseases, The Second Medical Center, Chinese PLA General Hospital, Beijing, China; ^4^Department of Critical Care Medicine, The 967th Hospital of Joint Logistics Support Force of Chinese PLA, Dalian, China; ^5^Department of Emergency, The 967th Hospital of Joint Logistics Support Force of Chinese PLA, Dalian, China

**Keywords:** atrial fibrillation, radiofrequency catheter ablation, antiarrhythmic drug, reactive hyperemia index, elderly, clinical outcome

## Abstract

**Background:**

Patients with atrial fibrillation (AF) who undergo radiofrequency catheter ablation (RFCA) necessitate the administration of antiarrhythmic drugs to prevent early recurrence. The clinical outcomes among these patients may be influenced by varying antiarrhythmic regimens.

**Objectives:**

To identify the risk factors associated with early recurrence and compare the clinical outcomes among different antiarrhythmic regimens in elderly patients with AF following radiofrequency catheter ablation (RFCA) during a 3-month period.

**Methods:**

A retrospective observational study encompassed 420 elderly patients with AF following RFCA. Baseline data were collected during the initial postoperative visit and clinical outcomes were carefully monitored over a 3-month follow-up period. Logistic regression and Cox-proportional hazard regression analyses were performed to investigate the relationship between various antiarrhythmic regimens and the clinical outcomes.

**Results:**

Multivariate logistic regression analysis revealed that age (*p* = 0.001), left atrial diameter (*p* < 0.001), left ventricular diameter (*p* = 0.015), reactive hyperemia index (RHI) (*p* < 0.001), antiarrhythmic drug (*p* < 0.001) and hs-cTnI (*p* = 0.017) were independent risk factors of early recurrence. Furthermore, in cox survival regression analysis model, survival rate of early recurrence in the amiodarone group was higher than in the propafenone group (HR 2.30, 95%CI 1.17–4.53, *p* = 0.016) and in the sotalol group (HR 3.60, 95%CI 2.17–5.95, *p* < 0.001). Compared to the amiodarone group, the incidence of liver dysfunction was lower in the dronedarone group (*p* = 0.046) and the propafenone group (*p* = 0.021). The incidence of bradyarrhythmia (*p* = 0.003), QT interval prolongation (*p* = 0.035) and atrioventricular transmission block (*p* = 0.021) were higher in the sotalol group than in the amiodarone group.

**Conclusion:**

RHI was identified as an independent risk factor for early recurrence among elderly AF patients after RFCA. Compared to amiodarone, propafenone and sotalol exhibited an elevated risk of early recurrence. Although there was no significant difference in early recurrence between amiodarone and dronedarone, dronedarone emerged as the preferred option due to its lower frequency of adverse drug reactions than amiodarone.

## Introduction

Atrial fibrillation (AF) is the most prevalent cardiac arrhythmia in clinical practice, posing an elevated risk of adverse cardiovascular and cerebrovascular outcomes (1, 2). When AF is accompanied by abnormal haemodynamics, it can lead to platelet activation, structural heart changes, and endothelial dysfunction or damage, ultimately increasing the risk of thrombogenesis (3). Effective management of AF’s abnormal heart rhythm can significantly reduce the occurrence of adverse clinical events (4). Middle-aged and elderly patients constitute the majority of adult patients with AF. Given the aging population and its evolving social structure, this trend will inevitably bring significant health burdens to society (5).

Radiofrequency catheter ablation (RFCA) has firmly established itself as a reliable rhythm control treatment for patients with AF, especially for persistent type with poor drug therapy effect (6). Recent findings have demonstrated that RFCA offers superior therapeutic outcomes compared to traditional antiarrhythmic drug (AAD) therapy alone (7). Moreover, endothelial function plays a crucial role in maintaining vascular homeostasis and preventing thromboembolic phenomena (8). RFCA could restore sinus rhythm and significantly improve vascular endothelial dysfunction caused by flow abnormalities, potentially conferring significant clinical benefits and improved prognosis for AF patients (9, 10).

Notably, patients undergoing RFCA face the risk for fibrillation recurrence. A range of factors, including age, heart size, and other conditions, have been associated with this risk (11, 12). Early recurrence is defined as the onset of atrial arrhythmia (AA) within 3 months after catheter ablation (known as the “blanking period”). In order to improve the effectiveness of RFCA, it is crucial to manage postoperative drug therapy during this critical period. This is because it can significantly impact left atrial (LA) anatomical and electrical remodeling, ultimately reducing the risk of AA recurrence (13). Given the availability of various AAD, different treatment regimens may lead to different outcomes (14).

However, there is currently a limited number of studies focusing on the prognosis of elderly patients with AF following RFCA. For this vulnerable patient population, it is imperative to conduct further analysis on the factors that contribute to early postoperative AF recurrence. Additionally, there is a significant lack of information regarding the efficacy and safety of ADDs in controlling early recurrence. Consequently, we conducted this present retrospective study to provide valuable insights on these pertinent issues and aimed to provide crucial clinical evidence for the prevention of AF among the elderly.

## Methods

### Study design and participants

We conducted a retrospective analysis of the clinical data of 420 elderly patients (aged 60 years and above) who had undergone RFCA for paroxysmal AF. These patients were followed up at the outpatient department of the 967th Hospital of Joint Logistics Support Force of Chinese PLA between 2018 and 2023. We excluded patients with histories of myocardial infarction, pulmonary dysfunction, thyroid dysfunction, hematologic disease, severe valvulopathy, severe liver and kidney dysfunction, malignant tumor, and New York Heart Association functional classifications above grade 2. Additionally, we excluded patients with incomplete clinical data, failed operations, or those who were unable to restore sinus rhythm. All patients had their clinical data collected within 1 week before ablation and were followed for a period of 3 months. The diagnosis of AF was based on the 2020 ESC/EACTS Guidelines for Diagnosis and Management of Atrial Fibrillation (15). We obtained written informed consent from all participants. This study conformed to the 1975 Declaration of Helsinki and was approved by the Human Ethics Committee of the 967th Hospital of Joint Logistics Support Force of Chinese PLA.

### Catheter ablation

The AAD discontinuation was recommended at least 1 month before the procedure. Sedation was achieved with a combination of midazolam and fentanyl throughout the electrophysiological study and ablation process. Endocardial 3D mapping was conducted using Biosense Webster’s CARTO mapping systems, facilitated by multipolar mapping catheters. For ablation, a 3.5-mm tip externally irrigated ablation catheter was utilized, ensuring a minimum target contact force exceeding 5 g. The ablation targets were determined based on achieving an Ablation Index of at least 400 on the posterior wall and 550 or higher on the anterior, superior, and inferior walls of the left atrium. This approach aimed to create ablation lesions of adequate quality during the wide-area circumferential ablation procedure. Pulmonary vein isolation (PVI) encompassed a comprehensive antral circumferential ablation procedure encompassing the pulmonary veins. The objective was to achieve electrical isolation, which was verified by identifying both entrance and exit block. PVI was further confirmed by the presence of spontaneous potentials or the complete absence of local electrograms, indicating no capture even at high-output pacing. Additional substrate modification, including linear ablation was left to the discretion of the operator and/or the attending physician.

### Data collection

All the data were obtained from standardized clinical electronic medical records, which included comprehensive information such as demographic details, comorbidities, nursing records, laboratory tests, echocardiography, electrocardiography (ECG), treatments, and clinical outcomes. The laboratory tests encompassed a range of assessments including routine blood tests, renal, liver, and thyroid function tests, coagulation profile, myocardial enzymes, electrolytes, and more. Abnormalities in laboratory findings were based on the hospital’s criteria. To assess endothelial function, we employed peripheral arterial tonometry using the EndoPAT2000 device (Itamar Medical, Caesarea, Israel) to measure the reactive hyperemia index (RHI). RHI quantifies the digital hyperemic response and impaired endothelial function was defined as a RHI less than 1.67. We measured these parameters on the day of admission, within 1 week before ablation.

### Antiarrhythmic drug therapy

Information about pre-medication were extracted from both electronic and traditional clinical medical records. Following the ablation procedure, all the patients were prescribed a 90-days use of AAD commencing on the day of the ablation (the recommended dosages were as follows: 50 mg/day for propafenone, 80 mg/day for sotalol, 800 mg/day for dronedarone, and 600 mg/day during the initial week, followed by a reduction to 200 mg/day for amiodarone). For safety reasons, the specific choice and dosage of AAD were determined by the attending physician.

### Definitions and endpoint

Paroxysmal AF was defined as an episode that spontaneously resolves or responds to AADs within 7 days of onset. Patients were prospectively followed for 3 months every 1 week via physician review. Patients were instructed to perform ECG at each visit, and instructed to present for ECG analysis if any symptoms occurred. With the usage of 24-ambulatory electrogram recorder (HCG-801, OMRON HEALTHCARE Co., Ltd.) once a month. The ambulatory electrogram recordings were then reviewed by cardiologists who were unaware of the treatment assignments. These cardiologists evaluated drug usage and the occurrence of AA recurrence. The clinical endpoint was defined as AA recurrence, which was characterized by AA lasting for more than 30 s or AA symptoms persisting during the blanking period (15).

### Statistical analysis

Statistical analyses were carried out using the latest version (27) of the SPSS software. Continuous variables were presented as median [inter quartile range (IQR)] and categorical variables were expressed as *n* (%). Mann–Whitney *U* test, Pearson *χ*^2^ test or Fisher’s exact test was employed to compare the differences between two groups as appropriate. To identify the risk factors, binary logistic regression models were used. Cox-proportional hazard regression analysis was used to investigate the association between risk factors and clinical endpoint. Variables that were considered clinically relevant or showed a univariate relationship with the outcomes (*p* < 0.10) were included in the multivariate regression model. To ensure parsimony of the final model, variables for inclusion were carefully chosen according to the number of events available. Odds ratio (OR) or hazard ratio (HR) was employed to determine relationships between risk factors and outcomes. A *p*-value of less than 0.05 was considered statistically significant.

## Results

A total of 420 AF patients who underwent RFCA were enrolled in this study between 2018 and 2023. Among them, there were 87 (20.7%) patients developed an early recurrence and they were divided into patients with early recurrence (*n* = 87) and patients without early recurrence (*n* = 333). Baseline characteristics of the two groups were listed in Table 1. Compared to patients without early recurrence, patients with early recurrence showed older age (*p* < 0.001), larger LA (*p* < 0.001) and left ventricular (*p* < 0.001) diameters, and lower ejection fraction (*p* < 0.001), and lower RHI (*p* < 0.001). As for blood biomedical tests, high-sensitivity C-reactive protein (hs-CRP) (*p* < 0.001), d-dimmer (*p* = 0.011), high-sensitivity cardiac troponin I (hs-cTnI) (*p* < 0.001), and brain natriuretic peptide (BNP) (*p* < 0.001) were higher in patients with early recurrence than patients without early recurrence.

**Table 1:** Baseline characteristics of elderly AF patients after RFCA.

	Total (*n* = 420)	With early recurrence (*n* = 87)	Without early recurrence (*n* = 333)	*p-*value
Age, years	68.00 (65.00–72.00)	70.00 (66.00–74.00)	67.00 (65.00–71.00)	<0.001
Males, *n* (%)	248 (59.0%)	51 (58.6%)	197 (59.2%)	0.928
BMI, kg/m^2^	25.30 (23.20–27.58)	25.30 (23.10–26.90)	25.40 (23.30–27.70)	0.356
Current smoker, *n* (%)	146 (34.8%)	30 (34.5%)	116 (34.8%)	0.951
Current drinker, *n* (%)	154 (36.7%)	34 (39.1%)	120 (36.0%)	0.600
Left atrial diameter, mm,	41.00 (36.00–46.00)	46.00 (41.00–53.00)	41.00 (36.00–44.00)	<0.001
Left ventricular diameter, mm,	46.00 (43.00–51.00)	50.00 (45.00–58.00)	46.00 (43.00–49.00)	<0.001
Ejection fraction, %	59.00 (53.00–63.00)	53.00 (41.00–61.00)	60.00 (55.00–64.00)	<0.001
Reactive hyperemia index	1.47 (1.20–1.74)	1.26 (0.98–1.48)	1.54 (1.28–1.80)	<0.001
Antiarrhythmic drug				
Propafenone, *n* (%)	49 (11.7%)	15 (17.2%)	34 (10.2%)	0.069
Sotalol, *n* (%)	137 (32.6%)	43 (49.4%)	94 (28.2%)	<0.001
Amiodarone, *n* (%)	206 (49.0%)	25 (28.7%)	181 (54.4%)	<0.001
Dronedarone, *n* (%)	28 (7.2%)	4 (6.7%)	24 (7.2%)	0.385
History				
Hypertension, *n* (%)	298 (71.0%)	64 (73.6%)	234 (70.3%)	0.547
Diabetes, *n* (%)	137 (32.6%)	26 (29.9%)	111 (33.3%)	0.837
Coronary heart disease, *n* (%)	262 (61.3%)	58 (66.7%)	204 (62.4%)	0.354
Valvular heart disease, *n* (%)	6 (1.4%)	2 (2.3%)	4 (1.2%)	0.794
Cardiomyopathy, *n* (%)	28 (6.7%)	7 (8.0%)	21 (6.3%)	0.562
Ischemic stroke, *n* (%)	40 (9.5%)	7 (8.0%)	33 (9.9%)	0.598
Peripheral vascular disease, *n* (%)	154 (45.0%)	35 (40.2%)	189 (46.2%)	0.315
Previous liver insufficiency, *n* (%)	3 (0.7%)	0 (0.0%)	3 (0.9%)	1.000
Previous renal insufficiency, *n* (%)	4 (1.0%)	1 (1.1%)	3 (0.9%)	1.000
Hyperlipemia, *n* (%)	97 (23.1%)	21 (24.1%)	76 (22.8%)	0.796
Hyperuricemia, *n* (%)	12 (2.9%)	2 (2.3%)	10 (3.0%)	1.000
Biomedical indicators				
Neutrophil percentage, *n* (%)	57.00 (52.13–63.13)	57.50 (52.80–64.90)	57.00 (52.00–62.70)	0.325
Lymphocyte percentage, *n* (%)	31.20 (25.53–36.50)	30.80 (24.70–36.50)	31.40 (25.65–36.50)	0.592
Leucocyte, 10^9^/L	6.04 (4.93–7.19)	6.42 (5.17–7.39)	5.96 (4.91–7.17)	0.237
Hemoglobin, g/L	135.00 (123.00–148.00)	137.00 (123.00–149.00)	135.00 (123.00–147.50)	0.442
Platelets, 10^9^/L	207.00 (170.25–246.75)	212.00 (172.00–248.00)	207.00 (170.00–246.00)	0.648
Hs-CRP, mg/dl	0.10 (0.10–0.28)	0.19 (0.10–0.56)	0.10 (0.10–0.22)	<0.001
Fasting blood glucose, mmol/L	5.04 (4.53–6.05)	5.17 (4.54–6.27)	5.02 (4.53–6.01)	0.357
Glycosylated hemoglobin, %	5.90 (5.60–6.60)	6.00 (5.60–6.70)	5.90 (5.55–6.60)	0.845
Creatinine, μmol/L	74.0 (64.10–88.40)	75.70 (63.70–88.40)	73.30 (64.10–88.85)	0.640
Uric acid, umol/L	333.30 (265.83–408.08)	350.70 (270.20–397.20)	330.20 (265.47–410.6)	0.655
Potassium, mmol/L	3.75 (3.53–3.99)	3.77 (3.50–4.00)	3.75 (3.54–3.99)	0.986
Sodium, mmol/L	140.20 (138.53–141.80)	140.50 (138.60–142.10)	140.20 (138.50–141.70)	0.207
Total cholesterol, mmol/L	3.85 (3.18–4.58)	3.82 (3.11–4.52)	3.85 (3.22–4.60)	0.434
Triglyceride, mmol/L	1.27 (0.93–1.81)	1.32 (0.94–2.06)	1.26 (0.93–1.78)	0.373
HDL-C, mmol/L	1.08 (0.92–1.29)	1.05 (0.92–1.25)	1.09 (0.92–1.32)	0.349
LDL-C, mmol/L	2.30 (1.73–2.87)	2.26 (1.61–2.82)	2.31 (1.74–2.94)	0.352
D-dimmer, ug/mL	0.24 (0.21–0.47)	0.33 (0.22–0.58)	0.23 (0.20–0.41)	0.011
ALT, U/L	22.25 (14.10–35.38)	19.80 (13.40–38.20)	22.60 (14.70–35.10)	0.163
AST, U/L	18.87 (15.20–24.6)	18.20 (14.90–23.10)	19.00 (15.43–24.65)	0.402
Prothrombin time, s	14.38 (13.44–15.49)	14.50 (13.70–15.80)	14.31 (13.40–15.38)	0.184
Activated partial thromboplastin time, s	33.45 (30.19–37.28)	34.30 (31.42–38.00)	33.38 (29.85–37.00)	0.087
INR	1.30 (1.14–1.51)	1.29 (1.11–1.48)	1.30 (1.16–1.52)	0.733
Creatine kinase-MB, IU/L	8.80 (7.10–11.28)	9.20 (7.40–12.00)	8.80 (7.05–11.10)	0.274
Hs-cTnI, ng/L	10.00 (10.00–12.00)	11.00 (10.00–21.00)	10.00 (10.00–10.00)	<0.001
Brain natriuretic peptide, pg./mL	39.74 (20.59–83.89)	75.20 (126.80–231.07)	34.61 (18.93–65.34)	<0.001

Data were expressed as *n* (%) and median (IQR); IQR, Interquartile range; *P-*value, Mann–Whitney *U* test or Pearson Chi-Square test. AF, atrial fibrillation; ALT, alanine aminotransferase; AST, aspartate aminotransferase; BMI, body mass index; HDL-C, high-density lipoprotein cholesterol; Hs-CRP, high-sensitivity C-reactive protein; Hs-cTnI, high-sensitivity cardiac troponin I; INR, international normalized ratio; LDL-C, low-density lipoprotein cholesterol; RFCA, radiofrequency catheter ablation.

We performed binary logistic regression models to find the risk factors of early recurrence. After calibration analysis, multivariate model showed that age (*p* = 0.001), LA diameter (*p* < 0.001), left ventricular diameter (*p* = 0.015), RHI (*p* < 0.001), AAD regimen (*p* < 0.001) and hs-cTnI (*p* = 0.017) were independent risk factors that affected the clinical outcome (Table 2).

**Table 2:** Risk factors of early recurrence in elderly AF patients after RFCA.

Variable	Univariable OR (95% CI)	*p-*value	Multivariable OR (95% CI)	*p-*value
Age, years	1.07 (1.03–1.12)	<0.001	1.10 (1.04–1.16)	0.001
Gender (male vs. female)	0.98 (0.61–1.58)	0.928		
BMI, kg/m^2^	0.97 (0.90–1.05)	0.456		
Current smoking	0.99 (0.60–1.62)	0.951		
Current drinking	1.14 (0.70–1.85)	0.225		
Left atrial diameter	1.13 (1.09–1.18)	<0.001	1.13 (1.07–1.20)	<0.001
Left ventricular diameter	1.11 (1.07–1.15)	<0.001	1.07 (1.01–1.14)	0.015
Ejection fraction	0.92 (0.90–0.95)	<0.001	1.00 (0.92–1.10)	0.945
Reactive hyperemia index	0.11 (0.06–0.23)	<0.001	0.06 (0.02–0.16)	<0.001
History				
Hypertension	1.18 (0.69–2.00)	0.547		
Diabetes	0.85 (0.51–1.42)	0.542		
Organic heart disease	1.50 (0.87–2.59)	0.146		
Ischemic stroke	0.80 (0.34–1.87)	0.599		
Peripheral vascular disease	0.78 (0.48–1.26)	0.316		
Previous liver insufficiency	-	0.999		
Previous kidney insufficiency	1.28 (0.13–12.45)	0.796		
Hyperlipemia	1.08 (0.62–1.87)	0.274		
Hyperuricemia	0.76 (0.16–3.53)	0.726		
Antiarrhythmic drug				
Amiodarone	ref	<0.001	ref	<0.001
Propafenone	3.19 (1.56–6.68)	0.002	4.23 (1.45–12.35)	0.008
Sotalol	3.31 (1.91–5.75)	<0.001	6.98 (3.26–14.93)	<0.001
Dronedarone	1.21 (0.39–3.77)	0.746	1.57 (0.40–6.15)	0.515
Biomedical indicators				
Leucocyte	1.05 (0.91–1.20)	0.501		
Hemoglobin	1.01 (0.99–1.02)	0.376		
Platelets	1.00 (1.00–1.01)	0.795		
Hs-CRP	1.64 (1.19–2.26)	0.003	1.35 (0.85–2.14)	0.199
Glycosylated hemoglobin	0.99 (0.82–1.20)	0.922		
Creatinine	1.00 (0.99–1.01)	0.771		
Uric acid	1.00 (1.00–1.00)	0.963		
Potassium	1.11 (0.59–2.09)	0.748		
Total cholesterol	0.92 (0.73–1.16)	0.493		
Triglyceride	1.15 (0.91–1.44)	0.241		
LDL-C	0.90 (0.69–1.17)	0.426		
ALT	0.99 (0.98–1.00)	0.138		
D-dimmer	1.08 (0.83–1.39)	0.572		
Prothrombin time	1.01 (0.92–1.10)	0.880		
Activated partial thromboplastin time	1.03 (1.00–1.07)	0.079	1.03 (0.98–1.09)	0.230
Hs-cTnI	1.07 (1.04–1.09)	<0.001	1.05 (1.01–1.08)	0.017
Brain natriuretic peptide	1.01 (1.01–1.01)	<0.001	1.00 (0.99–1.01)	0.984

95% CI, 95% confidence interval; OR, odds ratio; organic heart disease included coronary heart disease, valvular heart disease and cardiomyopathy. AF, atrial fibrillation; ALT, alanine aminotransferase; BMI, body mass index; Hs-CRP, high-sensitivity C-reactive protein; Hs-cTnI, high-sensitivity cardiac troponin I; LDL-C, low-density lipoprotein cholesterol; RFCA, radiofrequency catheter ablation.

**Figure 1:**
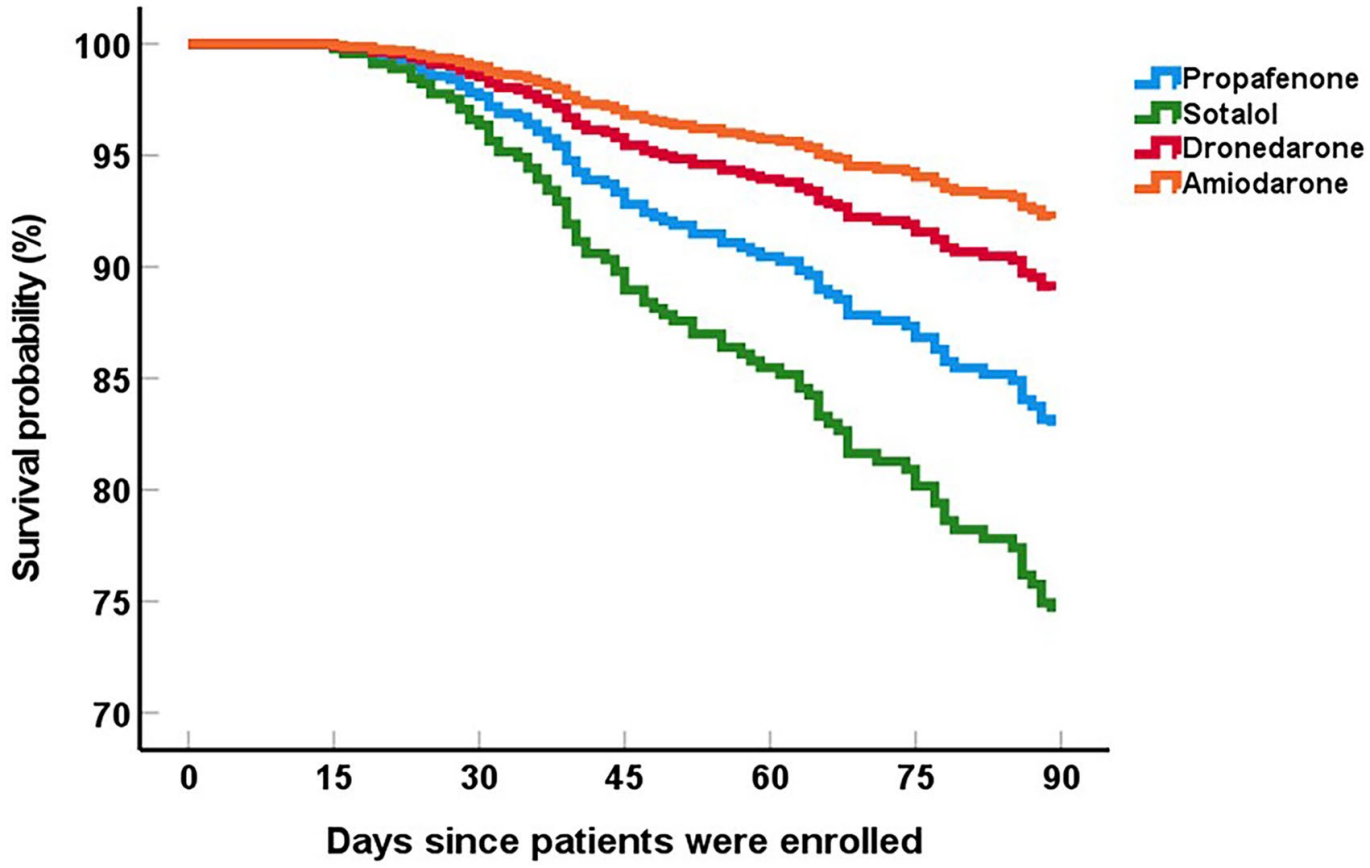
Event-free survival for early recurrence in elderly patients with AF after RFCA.

Sotalol was more used in patients with early recurrence (*p* < 0.001) while amiodarone was more used in patients without early recurrence (*p* < 0.001). Compared to patients without early recurrence, there seemed to be more propafenone usage in patients with early recurrence but the difference did not reach statistical significance. To investigate the effect of AAD regimen on clinical outcomes, we employed cox-proportional hazard regression model to compare early recurrence in the 3-month follow-up between the different groups. These patients were classified into four groups according to AAD application after RFCA. We observed that the survival rate of early recurrence in the amiodarone group was higher than in the propafenone group (HR 2.30, 95%CI 1.17–4.53, *p* = 0.016) and in the sotalol group (HR 3.60, 95%CI 2.17–5.95, *p* < 0.001). Besides, there was no difference of the survival rate of early recurrence between the amiodarone group and the dronedarone group (HR 1.43, 95%CI 0.49–4.17, *p* = 0.509) (Figure 1).

Moreover, we analyzed the occurrence of adverse reactions to different AAD regimens after RFCA. Compared to the amiodarone group, the incidence of liver dysfunction was lower in the dronedarone group (*p* = 0.046) and the propafenone group (*p* = 0.021). The incidence of rash/skin irritation was lower the propafenone group than in the amiodarone group (*p* = 0.036). Although the incidence of thyroid dysfunction was significantly lower in the sotalol group (*p* = 0.036), the incidence of bradyarrhythmia (*p* = 0.003), QT interval prolongation (*p* = 0.035) and atrioventricular transmission block (*p* = 0.021) were significantly increased than in the amiodarone group (Table 3).

**Table 3:** Adverse reactions to antiarrhythmic drugs of aged AF patients after RFCA.

	Total (*n* = 420)	Propafenone (*n* = 49)	Sotalol (*n* = 137)	Amiodarone (*n* = 206)	Dronedarone (*n* = 28)	P1	P2	P3
Renal dysfunction	16 (3.8%)	1 (2.0%)	5 (3.6%)	9 (4.4%)	0 (0.0%)	0.730	0.742	0.546
Liver dysfunction	51 (12.1%)	2 (4.1%)	16 (11.7%)	33 (16.0%)	0 (0.0%)	0.021	0.361	0.046
Thyroid dysfunction	18 (4.3%)	0 (0.0%)	0 (0.0%)	18 (8.7%)	0 (0.0%)	0.066	<0.001	0.211
Gastrointestinal symptoms	27 (6.4%)	2 (4.1%)	6 (4.4%)	20 (9.7%)	0 (0.0%)	0.328	0.068	0.173
Dizziness/headache	35 (8.3%)	5 (10.2%)	14 (10.2%)	16 (7.8%)	0 (0.0%)	0.577	0.431	0.259
Pulmonary abnormality	0 (0.0%)	0 (0.0%)	0 (0.0%)	0 (0.0%)	0 (0.0%)	–	–	–
Rash/skin irritation	43 (10.2%)	1 (2.0%)	17 (12.4%)	25 (12.1%)	0 (0.0%)	0.036	0.940	0.104
Bradyarrhythmia	33 (7.9%)	3 (6.1%)	19 (13.9%)	10 (4.9%)	1 (3.6%)	0.999	0.003	1.000
QT interval prolongation	7 (1.7%)	0 (0.0%)	6 (4.4%)	1 (0.5%)	0 (0.0%)	1.000	0.035	1.000
Atrioventricular transmission block	6 (1.4%)	1 (2.0%)	5 (2.9%)	0 (0.0%)	0 (0.0%)	0.192	0.021	–

*P-*value, Pearson Chi-Square test, Continuity correction test or Fisher’s exact test; P1, propafenone vs. amiodarone; P2, sotalol vs. amiodarone; P1, dronedarone vs. amiodarone. AF, atrial fibrillation; RFCA, radiofrequency catheter ablation.

## Discussion

This observational retrospective study of 420 consecutive subjects firstly showed RHI was an independent risk factor of early recurrence in elderly patients with AF after RFCA. Different AAD regimens affected the clinical outcomes between of these patients. We found that amiodarone and dronedarone significantly reduced the risk of early recurrence after RFCA than propafenone and sotalol. Moreover, we supposed that dronedarone may be a better option because of its lower incidence of adverse drug reactions compared to amiodarone in elderly patients.

Atrial fibrillation is the most frequently encountered sustained tachyarrhythmia in clinical practice (16). RFCA has emerged as the most effective method for treating AF recently and it is the preferred choice to control cardiac rhythm in patients with drug-refractory symptomatic AF and tachycardia cardiomyopathy AF (17, 18). However, postoperative recurrence remains a significant challenge. Early postoperative recurrence of RFCA refers to any AA lasting ≥30s within 3 months after ablation (19, 20). Effective prediction of early recurrence can guide timely intervention and prevent long-term recurrence, ultimately improving patient outcomes (21). Multiple factors have been reported could affect recurrence after RFCA, including age, gender, baseline cardiac function, and heart size (11, 12, 22). Even rare factors like anxiety have been demonstrated to be linked with it (23). Besides, other factors such as estimated glomerular filtration rate, BNP, soluble tumorigenic inhibition 2 and serum cholesterol have also been demonstrated associated with AF recurrence post-ablation (24–27). Our results were basically consistent with previous findings. However, we employed a non-invasive, highly reproducible method refereed as RHI to assess endothelial function in our study, and found RHI served as an independent risk factor for AF recurrence after ablation which exhibiting good predictive efficacy. This added information to the effective prediction of postoperative AF early recurrence.

Endothelial function is crucial for physiological activities. Patients with AF exhibit significantly reduced endothelial function compared to those with normal sinus rhythm, which is associated with adverse cardiovascular outcomes (28, 29). Moreover, AF triggers the elevation of c-reactive protein and cytokines, exerting a proinflammatory effect on endothelial cells, potentially leading to endothelial dysfunction (30). Additionally, patients with AF are generally older and often have other co-morbidities such as hypertension and diabetes, all of which could cause endothelial damage (31). Impaired endothelial function can also lead to abnormal hemodynamic changes, exacerbating or inducing AF, potentially perpetuating a vicious cycle leading to worse endothelial dysfunction and persistent AF (32). However, endothelial dysfunction can be reversed through various interventions, including dietary modifications, exercise, and drug administration (33). This present study confirmed the potential association between endothelial function and AF early recurrence after RFCA. Previous study has pointed out that assessing endothelial function (RHI) could enable risk stratification for cardiovascular events following AF ablation (34). Our results further emphasized the importance of assessing endothelial function prior to ablation procedures.

The application of AAD during the blanking period after ablation can promote the reversal of electrical remodeling in the atrium and stabilize the heart toward stable sinus rhythm without arrhythmia recurrence (35). This approach is suggested to not only effectively minimize the risk of recurrent AA but also to prevent late recurrences (36). However, other study pointed that AAD usage did not lead to improved clinical outcomes in the later stages (37). Given the high cost and potential postprocedural complications of repeat ablation, promoting sinus rhythm post-ablation through the administration of AAD is still essential. However, consensus is lacking regarding the optimal selection, dosage, and duration of AAD treatment after ablation due to existing AADs have limited safety and effectiveness (15, 38). Additionally, elderly patients account for a higher incidence rate of AF (5, 39). Currently, there is still limited information available on the optimal ADD treatment regimen for this special population following RFCA. Study have demonstrated class III antiarrhythmic drugs exhibiting a more pronounced effect (40). As a multi-ion channel blocker AAD, amiodarone is currently the most widely utilized AAD for the maintenance of sinus rhythm. It also plays a role in controlling atrial inflammation and correcting cardiac autonomic nervous dysfunction (41–43). Similar to our results, compared to propafenone and sotalol, it was more effective in maintaining sinus rhythm (41, 44).

However, it is crucial to remain vigilant about the potential side effects of prolonged AAD usage especially in elderly patients. Amiodarone’s long half-life and tissue accumulation can potentially lead to multiple adverse drug reactions with prolonged use (40). Sotalol has a high risk of cardiotoxicity (44). Besides, more arrhythmias occurred when sotalol was maintained at 80 mg/d within 3 months after RFCA in our study. Dronedarone, a benzofuran derivative, exhibits electrophysiological characteristics belonging to all four Vaughan-Williams classes. It lacks the iodine moiety and has a methane sulfonyl group that reduces its lipophilicity and tissue accumulation potential. Thus, it held favorable safety profile of dronedarone in patients with AF and its lower risk of amiodarone-like organ toxicity (45). However, dronedarone showed lower efficacy in maintaining sinus rhythm in AF patients after electrical cardioversion compared to amiodarone (46). Our findings indicated that dronedarone exhibited comparable efficacy to amiodarone in controlling early recurrence after ablation in elderly patients with AF. However, dronedarone demonstrated a lower incidence of adverse events, suggesting that it could be a preferential choice. The disparity could be attributed to differences in the patient population included, and further confirmation is required in the future.

## Limitations

This study has several limitations. Firstly, despite enrolling patients over nearly six consecutive years, the sample size remained relatively small. Secondly, the study was conducted at a single center, which may have resulted in a limited variety of patients enrolled. Thirdly, there was a notable absence of comprehensive analysis pertaining to the confounding factors encountered during RFCA procedure and subsequent postoperative follow-up period. These factors encompass subtle variations in surgical techniques, individualized differences in disease presentation (specifically COVID-19), disparities in the preoperative cessation period of ADDs, as well as biases in medication selection influenced by the clinician’s clinical experience. All of these could potentially affect the clinical prognosis. Lastly, due to disparities in baseline clinical characteristics that could have biased our results, we attempted to adjust for these differences through a multivariable analysis encompassing a broad range of variables. However, given the absence of a rigorous matching criterion, the findings of this retrospective study should be interpreted with caution.

## Conclusion

In summary, the findings of the present analysis revealed that RHI served as an independent predictor of early recurrence in elderly patients with AF after RFCA. Notably, amiodarone and dronedarone were found to be more effective in reducing the risk of early recurrence compared to propafenone and sotalol. Furthermore, we hypothesized that dronedarone might be a preferable choice given its relatively lower incidence of adverse drug reactions compared to amiodarone.

## Data availability statement

The original contributions presented in the study are included in the article/supplementary material, further inquiries can be directed to the corresponding authors.

## Ethics statement

The studies involving humans were approved by Human Ethics Committee of the 967th Hospital of Joint Logistics Support Force of Chinese PLA (No. PLA967-GC2023-21). The studies were conducted in accordance with the local legislation and institutional requirements. The participants provided their written informed consent to participate in this study.

## Author contributions

CF: Writing – review & editing, Software, Methodology, Investigation, Formal analysis, Data curation. CZ: Writing – review & editing, Software, Methodology, Investigation, Formal analysis, Data curation. YM: Writing – review & editing, Software, Methodology, Investigation, Formal analysis, Data curation. YL: Writing – review & editing, Validation, Resources, Project administration, Funding acquisition. RC: Writing – original draft, Methodology, Conceptualization. HZ: Writing – review & editing, Visualization, Validation, Supervision, Resources, Project administration, Conceptualization.
